# Developmental Angiogenesis Requires the Mitochondrial Phenylalanyl-tRNA Synthetase

**DOI:** 10.3389/fcvm.2021.724846

**Published:** 2021-09-01

**Authors:** Bowen Li, Kun Chen, Fangfang Liu, Juan Zhang, Xihui Chen, Tangdong Chen, Qi Chen, Yan Yao, Weihong Hu, Li Wang, Yuanming Wu

**Affiliations:** ^1^Department of Biochemistry and Molecular Biology, Air Force Medical University, Xi'an, China; ^2^Shaanxi Provincial Key Laboratory of Clinic Genetics, Air Force Medical University, Xi'an, China; ^3^Department of Anatomy, Histology and Embryology and K.K. Leung Brain Research Centre, Air Force Medical University, Xi'an, China; ^4^Department of Neurosciences, Air Force Medical University, Xi'an, China; ^5^Department of Biochemistry and Molecular Biology, College of Life Sciences, Northwest University, Xi'an, China; ^6^Department of Clinical Medicine, Yan'an University, Yan'an, China; ^7^School of Aerospace Medicine, Air Force Medical University, Xi'an, China

**Keywords:** mtARSs, FARS2, mitochondrial dysfunction, angiogenesis, zebrafish, HUVECs

## Abstract

**Background:** Mitochondrial aminoacyl-tRNA synthetases (mtARSs) catalyze the binding of specific amino acids to their cognate tRNAs and play an essential role in the synthesis of proteins encoded by mitochondrial DNA. Defects in mtARSs have been linked to human diseases, but their tissue-specific pathophysiology remains elusive. Here we examined the role of mitochondrial phenylalanyl-tRNA synthetase (FARS2) in developmental angiogenesis and its potential contribution to the pathogenesis of cardiovascular disease.

**Methods:** Morpholinos were injected into fertilized zebrafish ova to establish an *in vivo fars2* knock-down model. A visualization of the vasculature was achieved by using *Tg (fli1: EGFP)* ^*y*1^ transgenic zebrafish. In addition, small interference RNAs (siRNAs) were transferred into human umbilical vein endothelial cells (HUVECs) to establish an *in vitro FARS2* knock-down model. Cell motility, proliferation, and tubulogenesis were determined using scratch-wound CCK8, transwell-based migration, and tube formation assays. In addition, mitochondria- and non-mitochondria-related respiration were evaluated using a Seahorse XF24 analyzer and flow cytometry assays. Analyses of the expression levels of transcripts and proteins were performed using qRT-PCR and western blotting, respectively.

**Results:** The knock-down of *fars2* hampered the embryonic development in zebrafish and delayed the formation of the vasculature in *Tg (fli1: EGFP)* ^*y*1^ transgenic zebrafish. In addition, the siRNA-mediated knock-down of *FARS2* impaired angiogenesis in HUVECs as indicated by decreased cell motility and tube formation capacity. The knock-down of *FARS2* also produced variable decreases in mitochondrial- and non-mitochondrial respiration in HUVECs and disrupted the regulatory pathways of angiogenesis in both HUVECs and zebrafish.

**Conclusion:** Our current work offers novel insights into angiogenesis defects and cardiovascular diseases induced by *FARS2* deficiency.

## Introduction

Aminoacyl-tRNA synthetases pair amino acids with their cognate tRNAs and are therefore crucial for protein synthesis (1). Human mitochondrial DNA (mtDNA) encodes 13 indispensable subunits of the oxidative phosphorylation system (OXPHOs), 22 tRNAs, and two rRNAs (2). Mitochondrial aminoacyl-tRNA synthetases (mtARSs) play an important role in the translation of mtDNA coding genes. Although deficiencies in mtARSs are naturally assumed to impact mitochondrial protein synthesis, they have also been associated with various tissue-specific phenotypes (3–5). In particular, mutations in several mtARSs result in encephalopathies (*RARS2, NARS2, CARS2, IARS2, FARS2, PARS2, TARS2*, and *VARS2*), leukodystrophies (*AARS2, DARS2, EARS2*, and *MARS2*), or Perrault syndrome (*HARS2* and *LARS2*). In addition, mutations in three other aminoacyl-tRNA synthetases cause cardiomyopathies (*AARS2, GARS*, and *KARS*), while mutations in *YARS2* and *SARS2* cause MLASA syndrome and HUPRA syndrome, respectively. Notably, different clinical features have been reported in patients with mutations in the same mtARSs gene (5, 6).

Mitochondrial phenylalanyl-tRNA synthetase, encoded by the nuclear gene *FARS2*, catalyzes the recognition and binding of Phe and mt-tRNA^Phe^ in the mitochondria (5). Mutations in the *FARS2* gene are associated with central nervous system (CNS) diseases, such as autosomal recessive spastic paraplegia (7), epileptic encephalopathy (8–10), and infantile mitochondrial Alpers encephalopathy (11–13). In addition, our group reported that a missense homozygous mutation [c.424 G > T (p.D142Y)] in the *FARS2* gene was the underlying cause of hereditary spastic paraplegia in a Chinese family (7). Because CNS disorders are recognized as the major manifestations of *FARS2* gene mutations, previous research into the potential molecular mechanisms involved in the pathogenicity of these mutations has focused on the CNS (7, 8, 10, 12–16), and little is known about their effects on the cardiovascular system.

Cardiovascular diseases (CVDs), including stroke, heart failure, coronary artery disease, cardiomyopathy, and hypertensive heart disease, are some of the leading causes of death worldwide (17–19). Nonetheless, the etiology of CVDs has not been well investigated on account of their multi-factorial causes, covering inherited and environmental factors (20). Endothelial cells (ECs) play an indispensable role in angiogenesis and vascular remodeling, and endothelial dysfunction occurs in the early stages of CVDs such as coronary artery disease (21, 22). Angiogenesis, a process in which new blood vessels are formed from pre-existing vessels, is crucial for embryogenesis, tissue healing, and placental vascularization (23). In response to angiogenic stimuli, ECs differentiate into two distinct subtypes that perform characteristic functions: the tip cells extend the filopodia of the vascular branch frontlines, and the stalk cells extend the vascular branches behind the tip cells. Following the formation of the vascular network and blood perfusion, ECs are trans-differentiated into quiescent phalanx cells that line the new vessels (24–26). This complex process of EC specialization is regulated by multifarious signaling molecules, including paracrine and autocrine factors, as well as by oxidative respiratory metabolism. The mitochondria play an essential role in cellular oxidative respiration; however, although angiogenesis is an energy-intensive process, the respiratory metabolism in ECs is highly glycolytic and relies little on the mitochondria (27–31). Nonetheless, the mitochondria not only play a major role in aerobic oxidation but are also key intracellular structures that regulate several EC functions (32–34). While mitochondria-related metabolism resulting from angiogenic stimuli has been studied extensively (34, 35), the functions of mitochondrial protein synthesis in angiogenesis are only partially understood.

Angiogenesis is regulated by a complex network of molecules. As one of the indispensable pathways regulating embryonic development, the Wnt signaling pathway regulates a variety of complex biological processes (36, 37). The high expression levels of Wnt signaling genes in ECs during vasculature development support the pivotal role of this pathway in angiogenesis (38, 39). The Notch pathway, another evolutionarily conserved signaling system, is required for normal embryonic development, tissue homeostasis, and adult stem cell maintenance (40) and controls the specification of ECs in multiple vertebrates, such as chicken, zebrafish, and mice. Although the intracellular signaling pathways regulated by angiogenic stimulation have been investigated widely, the relationship between *FARS2* and signaling transduction in angiogenesis is unknown. Here, to determine whether the *FARS2* gene plays an essential role in developmental angiogenesis, we established two *FARS2* deficiency models. In the *in vivo* model, *Tg* (*fli1: EGFP*) ^*y*1^ transgenic zebrafish were treated with *fars2*-specific morpholinos (41). In the *in vitro* model, HUVECs were transfected with *FARS2*-specific small interference RNAs (siRNAs). By combining imaging, post-transcriptional manipulations of *FARS2*, and gene expression detection techniques, we found that FARS2 might participate in the pathological process of CVD by affecting the mitochondrial protein synthesis in ECs. Our data demonstrate a previously unanticipated role of *FARS2* in coordinating the angiogenic process.

## Materials and Methods

### Zebrafish Care and Maintenance

Adult wild-type AB strain zebrafish were maintained at 28.5 °C on a 14-h light/10-h dark cycle. Five to six pairs of zebrafish were set up for natural mating every time. On average, 200–300 embryos were generated. The embryos were maintained at 28.5 °C in fish water (0.2% Instant Ocean Salt in deionized water). The embryos were washed and staged according to (41). The establishment and characterization of *fli1a-EGFP* transgenic lines have been described elsewhere (42). The zebrafish facility at Shanghai Model Organisms Center is accredited by the Association for Assessment and Accreditation of Laboratory Animal Care International.

### Zebrafish Microinjections

Gene Tools, LLC (http://www.gene-tools.com/) designed the morpholinos (MOs). Antisense MOs (GeneTools) were microinjected into fertilized one-cell-stage embryos according to standard protocols (43). The sequences of the *fars2* translation-blocking and splice-blocking morpholinos were 5′-CATAGTAGCTGGTCCATAAGCCTCT-3′ (ATG-MO) and 5′-GAACATGGCAGGATTCCTACCTTCC-3′ (E3I3-MO), respectively. The sequence for the standard control morpholino was 5′-CCTCTTACCTCAGTTACAATTTATA-3′ (Gene Tools). The amount of the MOs used for injection was as follows: control-MO and ATG-MO, 4 ng per embryo; and E3I3-MO, 8 ng per embryo. Primers spanning *fars2* exon 2 (forward primer: 5′-CACTATCCCGTCTTCCATCAG-3′) and exon 4 (reverse primer: 5′-TGAAAGAACACCTCCATCTCG-3′) were used for RT-PCR analysis for confirmation of the efficacy of E3I3-MO. The primer *ef1*α sequences used as the internal control were 5′-GGAAATTCGAGACCAGCAAATAC-3′ (forward) and 5′-GATACCAGCCTCAAACTCACC-3′ (reverse).

### Quantitative Real-Time PCR

For zebrafish, total RNA was extracted from 30 to 50 embryos per group in Trizol (Roche) according to the instructions of the manufacturer. The RNA was reverse-transcribed using the PrimeScript RT reagent Kit with gDNA Eraser (Takara). The quantification of gene expression was performed in triplicates using Bio-rad iQ SYBR Green Supermix (Bio-rad) with detection on the Realplex system (Eppendorf). The relative gene expression quantification was based on the comparative threshold cycle method (2^−Δ*ΔCt*^) using *ef1*α as endogenous control gene. The primer sequences are given in Supplementary Table 1.

For HUVECs, total RNA was extracted from cells by using Axypre™ Multisource Total RNA Miniprep Kit (Axygen, cat. #365). The total RNA was reverse-transcribed with PrimeScript™ RT Master Mix (Takara, cat. #RR036A). Real-time fluorescent quantitative PCR was implemented by SYBR® Premix Ex Taq™ II (Takara, #RR820A) using 7500 system (Applied Biosystems). The procedures of the qRT-PCR were as follows: 95°C for 30 s for the first step and then for the ensuing 40 cycles–95 °C for 5 s and 60 °C for 30 s. Relative gene expression quantification was based on the comparative threshold cycle method (2^−ΔΔCt^) using *GAPDH* as the endogenous control gene (44). The primer sequences are given in Supplementary Table 1. All experiments were performed in triplicate and repeated three times independently.

### Zebrafish Angiogenesis Studies

To evaluate blood vessel formation in zebrafish, fertilized one-cell *fli1a-EGFP* transgenic line embryos were injected with *fars2*-MO and control-MO. At 48 hpf, the embryos were dechorionated and anesthetized with 0.016% MS-222 (tricaine methanesulfonate, Sigma-Aldrich, St. Louis, MO). The zebrafish were then oriented on the lateral side (anterior, left; posterior, right; dorsal, top) and mounted with 3% methylcellulose in a depression slide for observation by fluorescence microscopy. The phenotypes of complete intersegmental vessels (ISVs) [i.e., the number of ISVs that connect the dorsal anastomotic vessels to the dorsal longitudinal anastomotic vessels (DLAVs)], caudal vein plexus (CVP), DLAVs, and parachordal vessels (PAVs) were quantitatively analyzed. A total of 10 animals from at least three independent MO injections in each group were used in this experiment.

### Cell Culture and siRNA Transfection

Human umbilical vein endothelial cells (HUVECs, Sciencell cat. # 8000) were used from passages 3–9 and cultured in endothelial cell medium (ECM, Sciencell cat. # 1001) containing 500 ml of basal medium, 5% fetal bovine serum (FBS, Sciencell cat. #0025), 1% endothelial cell growth supplement (Sciencell cat. #1052), and 1% antibiotic solution (P/S, Sciencell cat. #0503) in 5% CO_2_ at 37°C. Then, 2 × 10^5^ cells, 10^5^ cells, and 10^4^ cells per well were seeded in six-well, 12-well, and 96-well plates for siRNA transfection. The cells were transfected with the following siRNAs: a FAM-labeled non-relevant control (50 nM), a non-relevant control (siCtrl, 50 nM), and *FARS2* siRNA (si-*FARS2*, 50 nM) from Ribobio™ (Guangzhou, China). The specific target sequences of these siRNAs are listed in Supplementary Table 2. X-tremeGENE siRNA Transfection Reagent (Roche) was used to build cells in the transfection process. In brief, X-tremeGENE siRNA Transfection Reagent and siRNA were separately diluted in Opti-MEM (Gibco cat. # 31985070) and mixed for 15 min at room temperature. Then, the mixture was added into the plates. The evaluation of transfection efficiency and functional assays on HUVECs was performed at 48 h after transfection. The transfection efficiency was monitored by calculating the percentage of FAM-positive cells under a fluorescence microscope.

### Western Blotting

We studied western blotting as described previously (44). The RIPA buffer (Biotime Biotechnology, cat. #P0013B, China), which included a protease inhibitor and a phosphatase inhibitor (Roche), was used for cell lysates. Then, the protein concentration was quantified by using a bicinchoninic acid (BCA) protein assay kit (Biovision, cat. #K813-2500). After mixing with 6 × loading buffer (Tiangen, cat#RT201), the protein samples were boiled for 10 mins in a metal bath for sufficient denaturation. Then, 10 μl (2 μg/μl) protein samples were measured in this study. After separating the different-molecular-weight proteins by 10% SDS-PAGE, all the proteins were transferred to the polyvinylidene difluoride membrane (Millipore, Germany, 0.45 μm) and blocked with 5% skim milk for 1 h at room temperature. The primary antibodies used included anti-FARS2 (1:1,000, Invitrogen), NOTCH1 (1:1,000, Abcam cat. #ab52627), β-catenin (1:1,000, Abcam cat. #ab16051), and GAPDH (1:20,000, Proteintech cat. #60004-1-Ig), followed by corresponding secondary antibodies (anti-mouse, 1:8,000 and anti-rabbit, 1:8,000, coupled to horseradish peroxidase). Proteins were revealed by chemiluminescence using the ECL kit (Millipore) (44). All experiments were performed in triplicate and repeated three times independently.

### Cell Proliferation and Transwell-Based Cell Migration Assays

The HUVEC cell proliferation assay *in vitro* was evaluated by CCK8 assay (44). The HUVECs were seeded in a 96-well plate with 100 μl ECM per well at 24 h before transfecting with siRNAs. Then, 10 μl CCK8 (HanBio, cat. # HB-CCK8-500T, Shanghai, China) reagent was added to each well for 1 h at 48 h after transfection with siRNAs. We measured the absorbance at 450 nm to detect proliferation of cells. All experiments were performed in triplicate and repeated three times independently.

The HUVEC migration assay was as described previously (45). In brief, for one well of a 24-well plate, the HUVECs transfected for 48 h were re-seeded in the upside of the transwell chamber (Corning) with 500 μl basal medium; 700 μl ECM (containing 5% FBS) was added in the bottom of the well. After cultivating for 24 h, the chamber was wiped with a cotton swab. The cells were fixed with 4% paraformaldehyde, stained with crystal violet solution, and counted under a microscope (× 20 objective). At least three different fields were averaged, and the experiment was repeated three times independently.

### Scratch-Wound Migration Assay

The HUVEC scratch-wound migration assay was evaluated by wound-healing assay (46). Briefly, the cells were transfected with siRNAs for 48 h (cultured upon reaching 90–95% confluence) in a six-well plate with 2 ml ECM; the HUVECs were scratched with the head of a 200-μl tip. The motility of the cells into the wound was imaged under a microscope (× 10 objective) at 0 and 6 h after wounding. The blank area in the wound was detected using Fiji Image J (NIH, Bethesda, MD, United States). All experiments were performed in triplicate and repeated three times independently.

### Tube Network Formation on a Matrigel Matrix

The method of tube network formation was studied as described previously (47). After transfection for 48 h, 300 μl of HUVEC suspension (4 × 10^5^ cells/ml) was re-seeded in a 24-well plate pre-coated with 289 μl Matrigel (10mg/ml, Corning cat. #354248) per well, which was polymerized by incubating in 37 °C for 30 min. Then, an Olympus microscope, with × 10 objectives, was used to take brightfield images of the 24-well plate. Fiji Image J (NIH, Bethesda, MD, United States) was employed to count the number of intersections in each field, and the total length of the structures was measured (48). At least three different fields were averaged, and the experiment was repeated three times independently.

### Mitochondrial Stress Testing Using Seahorse Technology

We studied mitochondrial stress testing as described previously (49). Seahorse Bioscience XFp extracellular flux analyzer (Agilent) was used to measure the mitochondrial stress test of HUVECs. This device works by creating a sealed chamber to measure oxygen consumption by the mitochondria in real time in the microplates under various stimuli. Mitochondrial reagents (Seahorse Bioscience Cell Mito Stress Test Kit, Agilent cat. #103010-100) were optimized at 2 μg/ml oligomycin (complex V inhibitor), 5 μM FCCP (a respiratory uncoupler), and 2 μM rotenone/antimycin A (inhibitors of complex I and complex III). A total of 30,000 HUVECs transfected with siRNAs for 48 h were seeded into the seahorse cell culture plate per well with 500 μl ECM and cultured at 37 °C in 5% CO_2_ humid atmosphere overnight. The sensor cartridge was incubated at 37 °C in a non-CO_2_ incubator for 24 h before detection. The cell culture plate and sensor cartridge were placed on XFp extracellular flux analyzer for Mito Stress Test. After detection, all the data were normalized to the BCA quantification of each well. This synthetic bioenergy spectrum provides detailed information on the various components of the respiratory chain. In brief, six essential parameters of mitochondrial respiration function were calculated from the results: basal respiration, ATP production, proton leakage, maximum respiration, spare respiration capacity, and non-mitochondrial respiration.

### Reactive Oxygen Species Assay

The intracellular reactive oxygen species (ROS) was analyzed by Reactive Oxygen Species Assay Kit (Beyotime cat. # S0033S, China). HUVECs (2 × 10^5^ per well of six-well plates) were seeded and transfected with siRNAs for 48 h. Then, the cells were washed with PBS once; 1 ml of DCFH-DA (1:1,000 dilution) was added to each collecting tube in the dark and incubated at 37 °C for 30 min. The labeled cells were collected and analyzed by flow cytometry at 488 nm. All experiments were repeated three times independently.

### Detection of ATP Levels

ATP dissolved in cells was detected by enhanced ATP assay kits (Beyotime cat. #S0027, China). According to the recommendations of the manufacturer, the standard curve was established and the concentration was detected by an enzyme reader (TECAN cat. #30086376, Switzerland). Finally, the ATP concentration was normalized by the BCA protein concentration method to eliminate the error caused by the difference of protein content. The quantification of the total ATP levels in HUVECs was conducted 48 h after transfection with siRNAs. All experiments were repeated three times independently.

### Image Acquisition

For zebrafish, embryos and larvae were analyzed with a Nikon SMZ 18 fluorescence microscope and subsequently photographed with digital cameras. A subset of images was adjusted for level, brightness, contrast, hue, and saturation with Adobe Photoshop 7.0 software (Adobe, San Jose, California) to optimally visualize the expression patterns. Quantitative image analyses was processed using image-based morphometric analysis (NIS-Elements D4.6, Japan) and Fiji Image J (NIH, Bethesda, MD, United States). Ten animals for each treatment were quantified, and the total signal per animal was averaged.

For HUVECs, all the experiment images were taken with an Olympus IX73 fluorescence microscope. Quantitative image analyses were processed using image Fiji Image J (NIH, Bethesda, MD, United States).

### Statistical Analysis

All data were presented as mean ± SEM. Statistical analysis and graphical representation of the data were performed using GraphPad Prism 8.3 (GraphPad Software, San Diego, CA). Statistical significance was performed using Student's *t*-test or ANOVA as appropriate. Statistical significance is indicated by an asterisk where *P* < 0.05; two asterisks, where *P* < 0.01; three asterisks, where *P* < 0.001; and four asterisks, where *P* < 0.0001.

## Results

### Expression of *fars2* Is Essential in the Early Stage of Zebrafish Embryo Development

**Figure 1:**
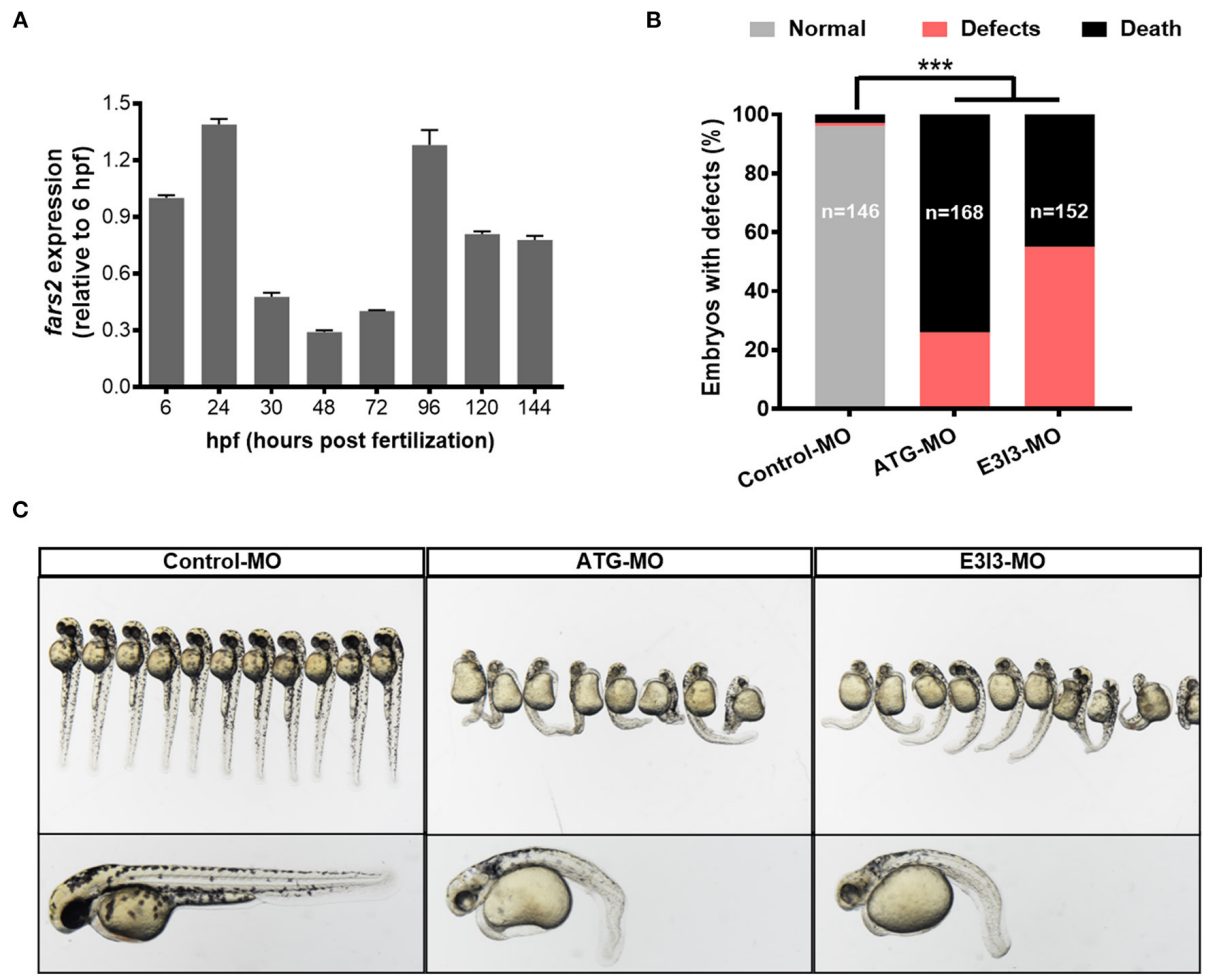
The expression of *fars2* is essential in the early stage of zebrafish embryo development. **(A)** The expression patterns of *fars2* during zebrafish embryonic development. The qRT-PCR analyses were performed at eight embryo development stages (6, 24, 30, 48, 72, 96, 120, and 144 hpf). **(B)** The percentages of embryos with developmental defects in zebrafish injected with a non-specific control or *fars2*-specific morpholinos (MOs). **(C)** Representative images of zebrafish at 50 hpf, following an injection with a non-specific control or *fars2*-specific MOs. ***P < 0.001.

Zebrafish (*Danio rerio*) is used extensively in angiogenesis studies because it undergoes rapid growth. The development of the vasculature in zebrafish can be divided into five major stages (50, 51). Compared with humans, the *fars2* gene was highly homologous, and the sequence similarity of Fars2 protein in zebrafish reached 71.39%. To explore its role in angiogenesis, we investigated the expression of *fars2* during the embryonic development of zebrafish. The qRT-PCR analyses of total embryos revealed that *fars2* transcription increased between 6 and 24 h post-fertilization (hpf) and then again between 72 and 96 hpf, which are the critical stages of vascular formation in zebrafish (Figure 1A). At 20 hpf, primary sprouts start to emerge bilaterally from the dorsal aorta at each vertical myoseptal boundary and then elongate dorsally, ramify, and interconnect along the dorsolateral roof of the neural tube to form paired dorsal longitudinal anastomotic vessels. The primary sprouts grow in a saltatory pattern, with numerous filopodia actively extending and retracting in all directions around the stretchy vessels (50, 52). The 3–6 days post-fertilization stage is the key period for the establishment of the systemic circulation in zebrafish embryos (50).

To investigate the role of *fars2* in zebrafish embryo development further, two specific MOs (ATG-MO and E3I3-MO) were designed to reduce its expression *in vivo* (Supplementary Figure 1A). Quantitative analyses performed after injecting one-cell fertilized ova with a non-specific control MO or the *fars2*-specific MOs confirmed the successful knock-down of *fars2* by the latter (Supplementary Figures 1B,C). Approximately 26.2% of *fars2* ATG morphants and 55.3% of E3I3 morphants presented an enlarged yolk sac, with the embryos displaying delayed growth and curved trunks (Figures 1B,C). The remaining embryos injected with the *fars2* MOs all died (Figure 1B).

Overall, these findings demonstrate that *fars2* is expressed at high levels during the critical period of angiogenesis in zebrafish and that the loss of *fars2* impairs embryonic development.

### Morpholino-Induced Knock-Down of *fars2* Delays Vascular Formation in Zebrafish

**Figure 2:**
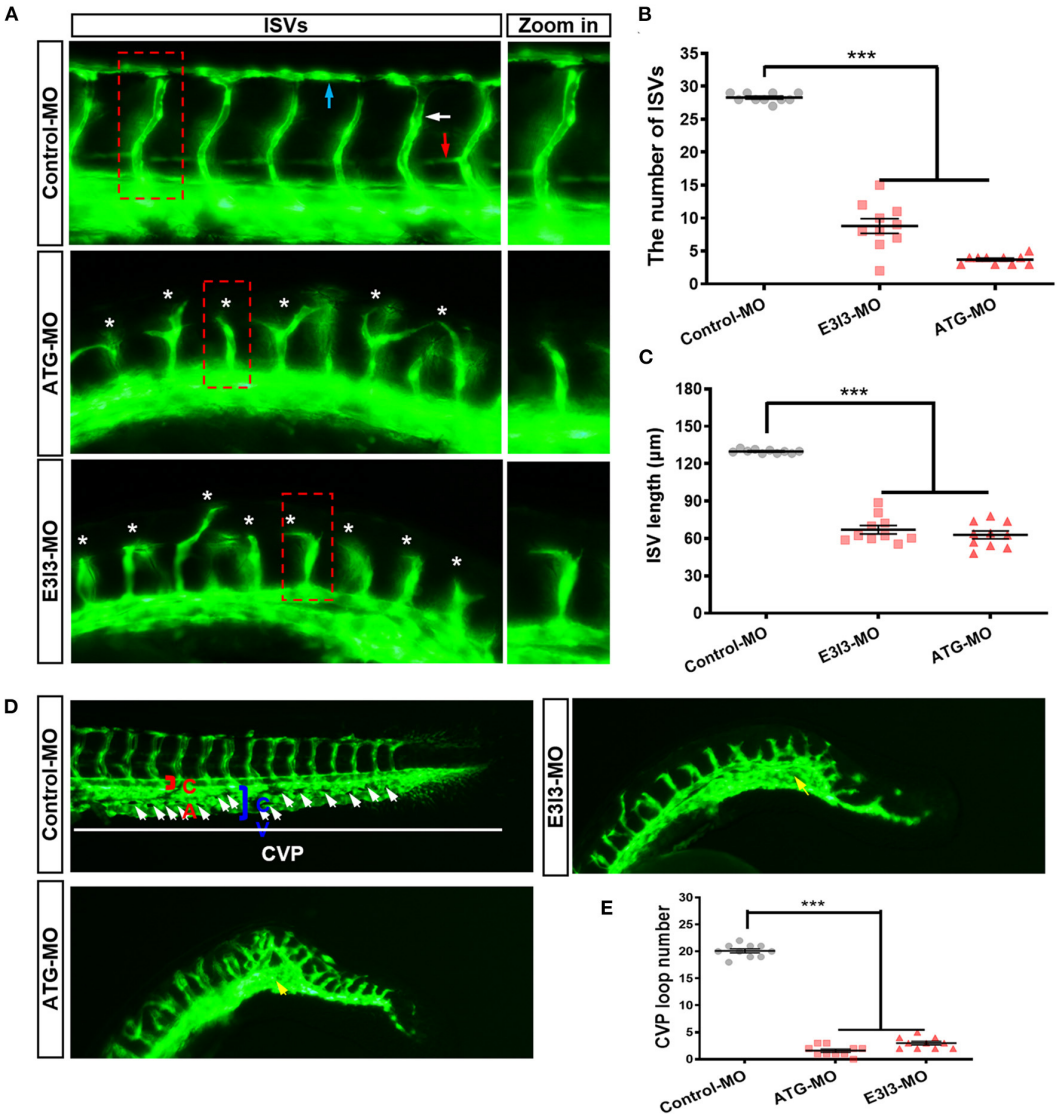
The morpholino-mediated knock-down of *fars2* delays vascular formation in zebrafish. **(A)** Representative images of the trunk regions of *Tg (fli1: EGFP)* ^*y*1^ embryos taken at 50 hpf. The intersegmental vessel (white arrow), dorsal longitudinal anastomotic vessel (blue arrow), parachordal vessel (red arrow), and ectopic sprouts (asterisks) are indicated. **(B,C)** The number of complete intersegmental vessels (ISVs) and the mean lengths of the ISVs in control and *fars2* morphants. The horizontal bars show the mean ± SEM (*n* = 10 per group). ^***^P < 0.001 *via* ANOVA. **(D)** Representative images of the caudal artery, caudal vein, and caudal vein plexus (CVP; arrows) in *Tg (fli1: EGFP)* ^*y*1^ embryos taken at 50 hpf. In the control embryo, the CVP formed a typical honeycomb structure in the tail (white arrows). The knock-down of *fars2* resulted in specific defects in CVP formation (yellow arrows). **(E)** Quantification of the loop number at the CVP. The horizontal bars show the mean ± SEM (*n* = 10 per group). ^***^P < 0.001 *via* ANOVA.

To examine its role in zebrafish developmental angiogenesis, *fars2* was knocked down in *Tg (fli1:EGFP)* ^*y*1^ transgenic zebrafish, which display a steady expression of EGFP within vascular ECs, allowing easy visualization of the vascular structures (41). The labeled ISVs and DLAVs showed regular development in the embryos injected with the control MO. By contrast, embryos injected with *fars2*-specific MOs displayed lower numbers of ISVs and ectopic sprouts (Figure 2A). The PAVs, the precursors to the lymphatic system, formed normally in control embryos, whereas *fars2* morphants displayed deficient PAV formation (Figure 2A). In addition, the number of complete ISVs (Figure 2B) and the mean length of ISVs (Figure 2C) were significantly lower in the *fars2* morphants than in the controls.

During zebrafish angiogenesis, new vessels that arise from axial veins and dorsal aortas form a primitive circulatory loop (53, 54). At 26–32 hpf, the posterior axial vein stretches ventrally and ultimately forms a “honeycomb-like” network named the CVP at 38 hpf. The shape of the CVP is produced by dorsal veins, ventral veins, and interlacing vessels (55, 56). In embryos injected with the control MO, the CVP formed canonical honeycomb-like structures at the tail at around 50 hpf. By contrast, *fars2* knock-down caused specific defects in CVP formation (Figure 2D). Furthermore, the number of loops at the CVP was lower in the *fars2* knock-down embryos than in the control embryos (Figure 2E). Overall, these findings demonstrate that MO-mediated knock-down of *fars2* disrupted the formation of ISVs, DLAVs, and the CVP during embryonic development in zebrafish.

### Deficiency of *FARS2* Impairs Cell Motility, Proliferation, Migration, and Tube Formation in HUVECs

To gain further insight into the function of *FARS2* in angiogenesis, we established an *in vitro FARS2* knock-down model using HUVECs and siRNAs. Western blot and qRT-PCR analyses confirmed the efficient knock-down of *FARS2* by three different siRNAs (si-*FARS2*). Compared with those in cells transfected with a control siRNA (siCtrl), the expression levels of the *FARS2* gene and protein were reduced by at least 30% following transfection with si-*FARS2* (Supplementary Figure 2).

**Figure 3:**
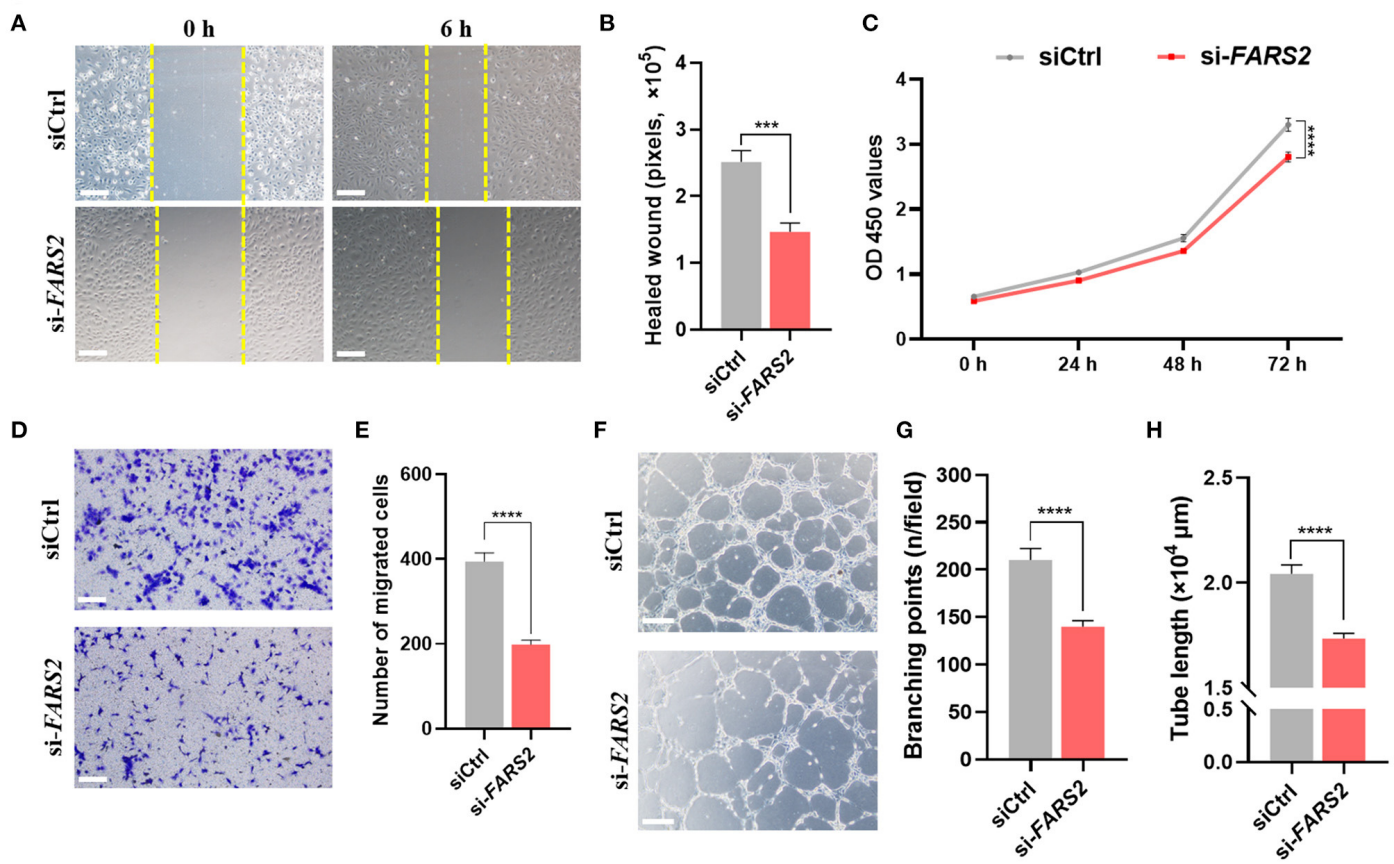
The deficiency of FARS2 impairs cell motility, proliferation, invasion, and tube formation in human umbilical vein endothelial cells (HUVECs). **(A)** Representative images of scratch-wound assays of HUVECs 0 and 6 h at 48 h after transfection with a control (siCtrl) or *FARS2*-specific (si-*FARS2*) siRNA. Scale bar = 200 μm. **(B)** Quantification of the healed wound area from **(A)**. Data are prepresented as the mean and SEM (*n* = 10). ^***^P < 0.001 *via* ANOVA. **(C)** A CCK8-based cell proliferation assay of HUVECs at the indicated time-points after transfection with siCtrl or si-*FARS2*. The measurements were made in triplicate (mean and SEM), and the results are indicative of three independent experiments. ^****^P < 0.0001. **(D)** Representative images of transwell-based migration assays of HUVECs 48 h after transfection with siCtrl or si-*FARS2*. Scale bar = 200 μm. **(E)** Quantification of the number of migrated cells from **(D)**. ^***^P < 0.001. **(F)** Representative images of tube network assays of HUVECs 48 h after transfection with siCtrl or si-*FARS2*. Scale bar = 200 μm. **(G,H)** Quantification of the branching points **(G)** and tube lengths **(H)** from **(F)**. The measurements were made in triplicate (mean and SEM), and the results are indicative of three independent experiments. ^****^P < 0.0001.

Scratch-wound assays, CCK8-based cell proliferation tests, and transwell-based migration assays revealed that the loss of FARS2 reduced the motility, proliferation, and migration capacity of HUVECs (Figures 3A–E). In addition, tubulogenesis was also reduced in cells transfected with si-*FARS2* (Figure 3F). Compared with those in cells transfected with siCtrl, the number of intersections in one field (Figure 3G) and the total length of the tube structures (Figure 3H) were lower following *FARS2* silencing. To our knowledge, this is the first report of an *in vitro FARS2* knock-down cell model created using siRNAs. Our findings demonstrate that the loss of FARS2 in HUVECs impairs cell motility, proliferation, invasion, and tube formation.

### *FARS2* Silencing Causes Mitochondrial Dysfunction in HUVECs

**Figure 4:**
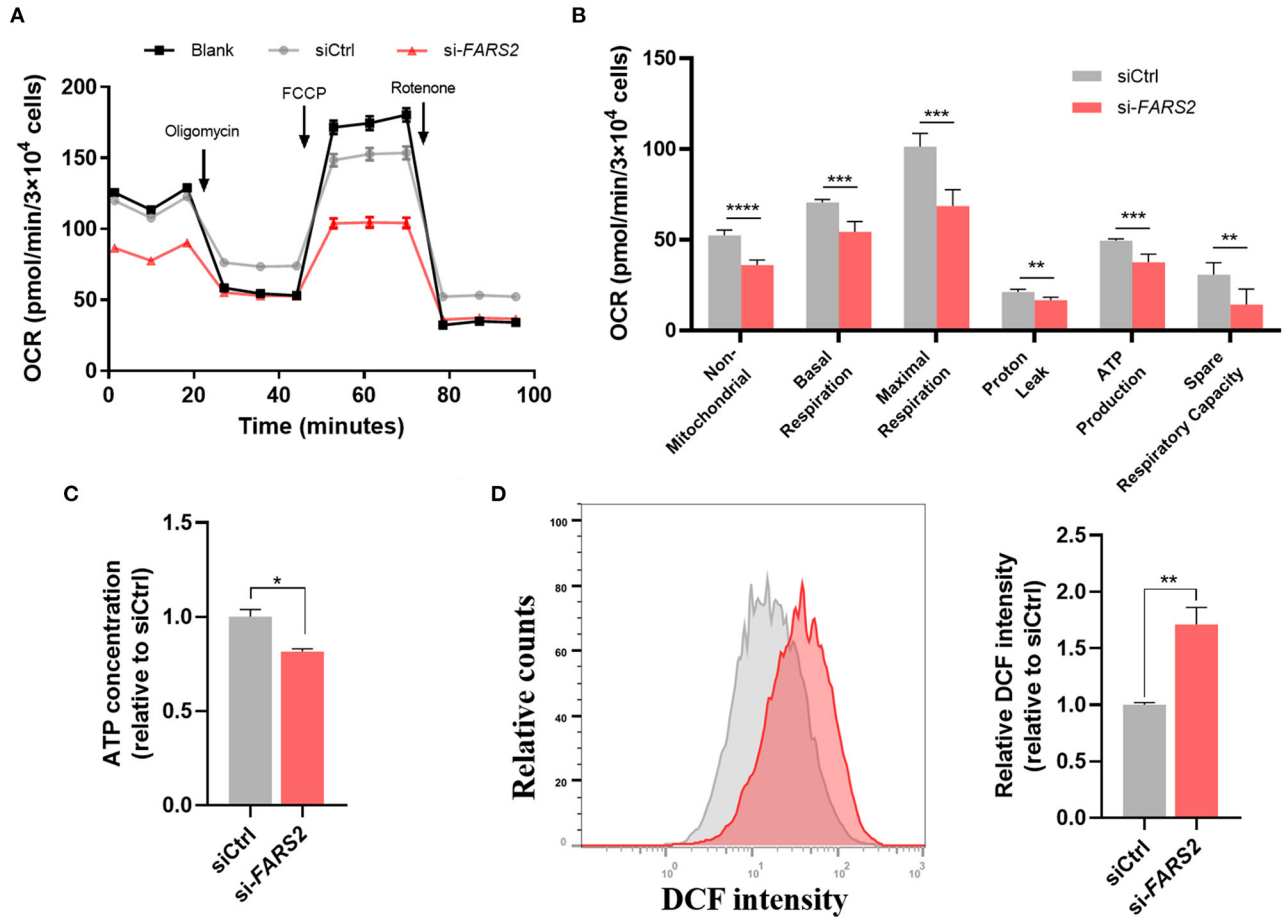
*FARS2* silencing causes mitochondrial dysfunction in human umbilical vein endothelial cells (HUVECs). **(A)** The oxygen consumption rate (OCR) in HUVECs transfected with siCtrl or si-*FARS2*. The HUVECs were seeded 48 h after transfection with siRNAs and 12 h before analysis using a Seahorse XF24 analyzer. The OCR was measured continuously throughout the experimental period, both at baseline and in the presence of the indicated drugs. **(B)** Non-mitochondrial respiration, basal respiration, maximal respiration, proton leak, ATP production, and spare respiratory capacity in control and FARS2-deficient HUVECs. The measurements were made in triplicate (mean and SEM). ^**^P < 0.01, ^***^P < 0.001, ^****^P < 0.0001. **(C)** The effects of FARS2 knock-down on intracellular reactive oxygen species production by HUVECs. The measurements were made in triplicate (mean and SEM). ^*^P < 0.05, **(D)** Quantification of total ATP levels in HUVECs 48 h after transfection with the indicated siRNAs. The measurements were made in triplicate (mean and SEM). ^**^P < 0.01.

As the *FARS2* gene encodes the mitochondrial phenylalanyl-tRNA synthetase, which is involved in the synthesis of mtDNA-coded OXPHOs subunits, we investigated mitochondrial respiration in HUVECs after *FARS2* silencing. To this end, a Seahorse Bioscience XF24 analyzer was used to measure the rates of non-mitochondrial respiration, basal respiration, maximal respiration, proton leak, ATP production, and spare respiratory capacity (57) in HUVECs transfected with siCtrl or si-*FARS2* for 48 h. Basal mitochondrial respiration, represented by the oxygen consumption rate (OCR), was lower in HUVECs transfected with si-*FARS2* than in non-transfected HUVECs or those transfected with siCtrl (Figures 4A,B). Following the addition of oligomycin, an inhibitor of ATP synthase, ATP production and proton leak were lower in si-*FARS2*-treated cells than in siCtrl-treated cells (Figure 4B). *FARS2* silencing also attenuated the OCR after the cells were treated with FCCP to maximize mitochondrial respiration (Figure 4B). In addition, after treatment with rotenone to uncouple the oxidation respiratory chain, the loss of FARS2 attenuated the OCR. Finally, the spare respiratory capacity, which was calculated based on the basal and maximal respiration values, was also lower in si-*FARS2*-treated cells than in siCtrl-treated cells (Figure 4B). The mitochondria produce ATP and are a main source of ROS. Reduced ATP production and increased levels of ROS are thought to occur as a result of mitochondrial dysfunction. Compared with the control cells, the FARS2-deficient HUVECs showed lower levels of sector ATP and increased levels of ROS (Figures 4C,D). Overall, these results suggest that silencing of the *FARS2* gene impairs mitochondria- and non-mitochondria-related respiration, leading to mitochondrial dysfunction in HUVECs.

### Deficiency of *FARS2* Impairs Angiogenesis by Disrupting the Notch and Wnt Signaling Pathways

**Figure 5:**
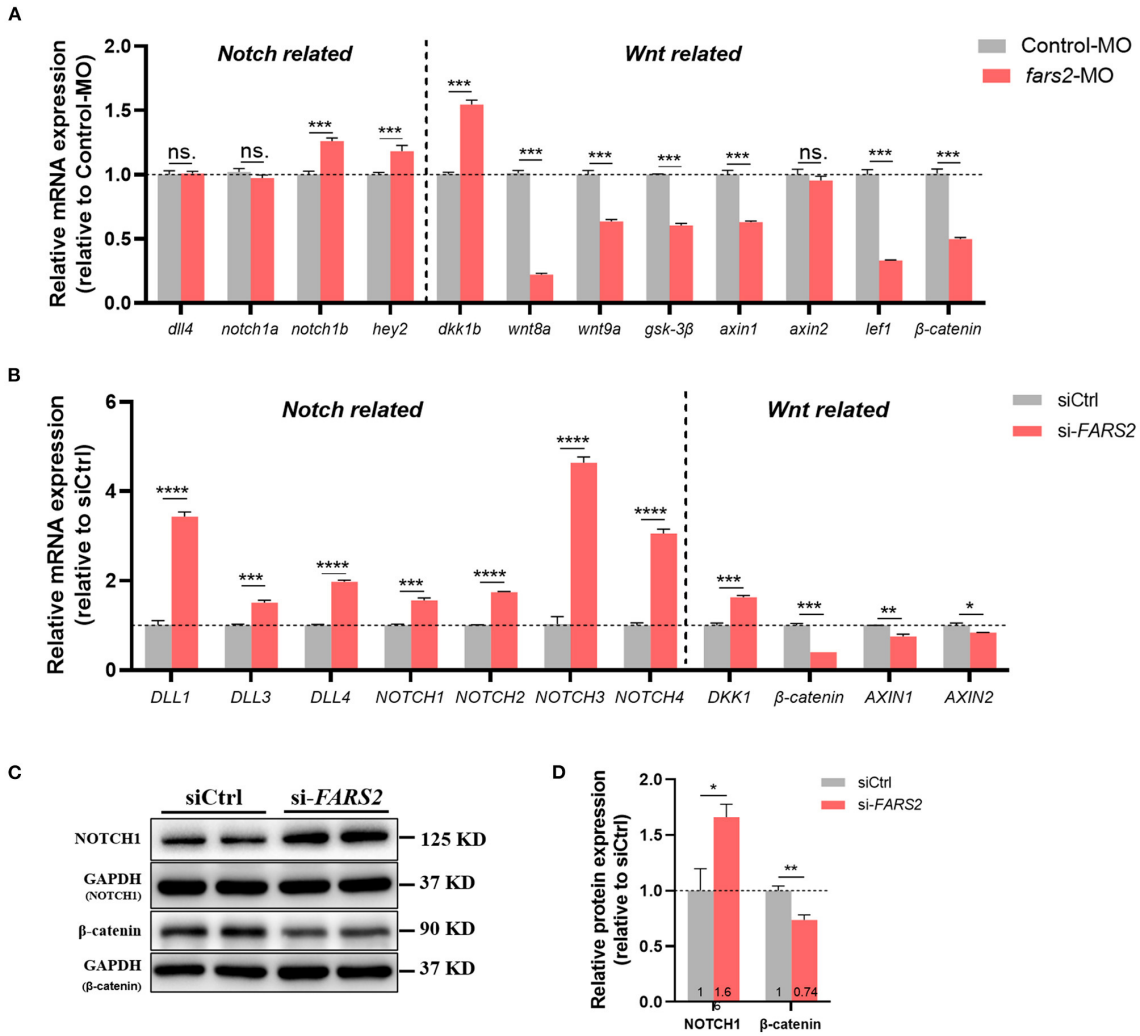
The deficiency of FARS2 impairs angiogenesis by disrupting the Notch and Wnt signaling pathways. **(A)** The expression levels of genes involved in the *Notch/Wnt* pathways in control and *fars2* zebrafish morphants, as determined by qRT-PCR analyses (*n* = 6–10 individual embryos). ^***^P < 0.001, ^**^P < 0.01, ^*^P < 0.05; ns, not significant. **(B)** The relative mRNA expression levels of *Notch/Wnt* pathway-related genes. The human umbilical vein endothelial cells (HUVECs) were transfected with the indicated siRNAs for 48 h and then harvested for qRT-PCR analysis. The measurements were made in triplicate (mean and SEM), and the results are indicative of three independent experiments. ^*^P < 0.05, ^**^P < 0.01, ^***^P < 0.001, ^****^P < 0.0001. **(C)** Western blot analyses of NOTCH1 and β-catenin protein levels. The HUVECs were transfected with the indicated siRNAs for 48 h prior to analysis. **(D)** Quantification of the western blotting data described in **(C)**. The measurements were made in triplicate (mean and SEM). ^*^P < 0.05, ^**^P < 0.01.

To explore the potential molecular mechanisms underlying the suppression of angiogenesis following MO-mediated knock-down of *fars2* in zebrafish, the expression levels of key genes in the Notch and Wnt pathways were examined using qRT-PCR. In zebrafish, *fars2* deficiency upregulated the *notch1b* (a Notch receptor) and *hey2* (a downstream gene in the Notch pathway) expression levels, indicating the activation of the Notch pathway (Figure 5A). In addition, *fars2* deficiency increased the expression level of *dkk1b* and decreased those of other downstream genes in the Wnt pathway, indicating an inhibition of Wnt signaling (Figure 5A).

As seen in zebrafish, siRNA-mediated knock-down of *FARS2* in HUVECs also activated the Notch signaling pathway by upregulating all four mammalian Notch receptors (*NOTCH1–4*) and three ligands (*DLL1, 3*, and *4*) to varying degrees (Figure 5B). In addition, the Wnt signaling pathway was inhibited after *FARS2* silencing, as indicated by the downregulation of Wnt downstream genes (β*-catenin, AXIN1*, and *AXIN2*) and upregulation of the Wnt signaling inhibitor gene *DKK1* (Figure 5B). Western blot analyses confirmed that the NOTCH1 and β-catenin protein levels were increased and decreased, respectively, following the siRNA-mediated knock-down of *FARS2* (Figures 5C,D). Overall, these findings demonstrate that the loss of FARS2 affects angiogenesis by disrupting the Notch and Wnt signaling pathways.

## Discussion

**Figure 6:**
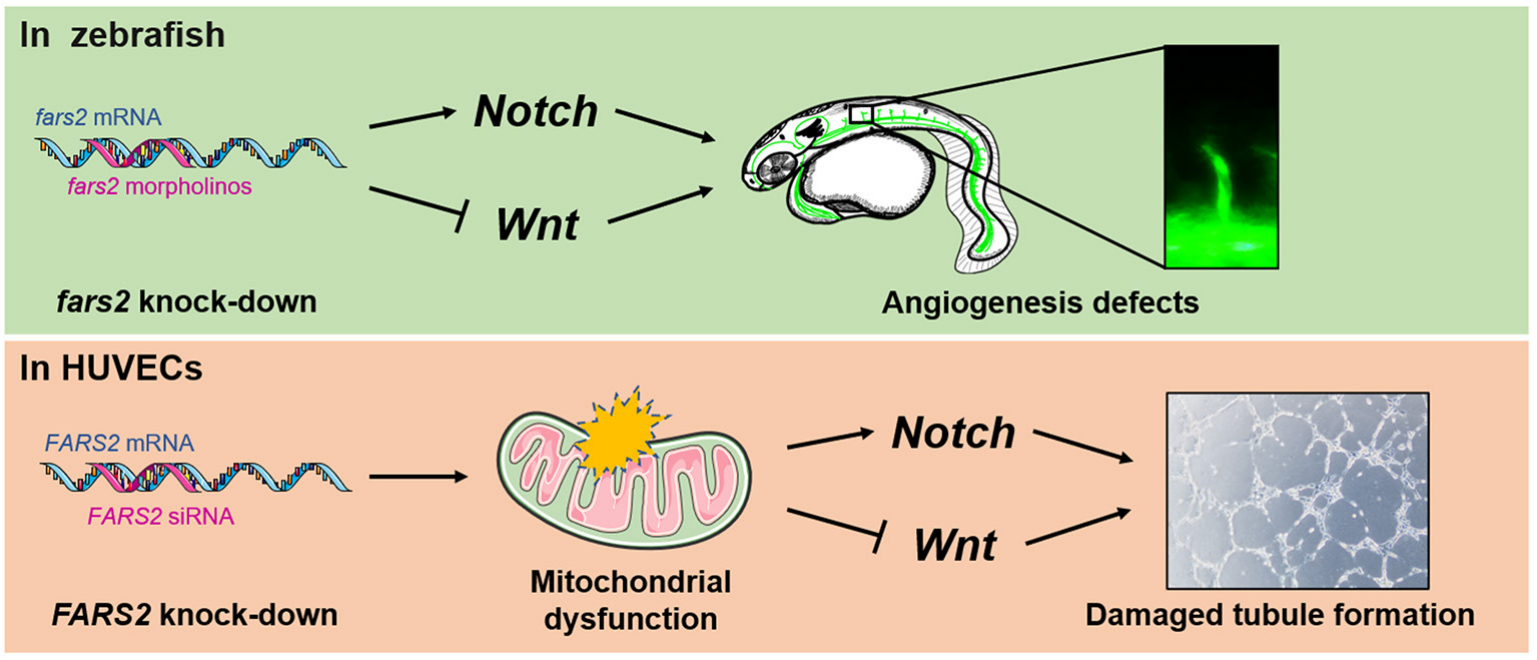
Developmental angiogenesis requires the mitochondrial phenylalanyl-tRNA synthetase. An overview of the mechanisms by which the deficiency of mitochondrial phenylalanyl-tRNA synthetase impairs angiogenesis by disrupting the *Notch/Wnt* pathways in zebrafish and human umbilical vein endothelial cells.

The results presented here show that mitochondrial phenylalanyl-tRNA synthetase plays an essential role in angiogenesis both *in vivo* and *in vitro*. Our initial analysis of the expression pattern of *fars2* during zebrafish embryonic development suggested that it plays a role in developmental angiogenesis. Subsequently, using MOs, we found that *fars2* deficiency caused the delayed development of zebrafish embryos and impaired vascular formation, including those of ISVs, DLAVs, PAVs, and the CVP. Similarly, we found that siRNA-mediated knock-down of *FARS2* in HUVECs impaired cell motility, proliferation, migration, and tube formation, confirming the role of FARS2 in angiogenesis. We also found that the loss of FARS2 led to mitochondrial dysfunction in HUVECs. Finally, we explored the possible mechanisms underlying the disruption of angiogenesis and found that *FARS2* deficiency may disrupt the Notch and Wnt signaling pathways, both of which are involved in angiogenesis (Figure 6).

The lethality of defects in ECs to mammalian embryos confirms the pivotal function of the vasculature in development. During embryonic development, two essential processes, vasculogenesis and angiogenesis, form the vasculature consisting of arterial, venous, and lymphatic vessels. Vasculogenesis is defined as the *de novo* emergence of vessels through the differentiation of angioblasts. Angiogenesis describes new vascular formation after the proliferation of ECs from pre-existing vessels (58–60). Much effort has been focused on investigating the key stages of vasculature development in mammalian embryos. The first sign of vascular formation occurs in the extraembryonic yolk sac blood island at the gastrulation stage as early as embryonic day 7.5. Subsequently, the blood island fuses to constitute the primary plexus, which leads to the establishment of the complex yolk sac vasculature (61, 62). Next, under the influence of complex transcriptional regulation and critical signaling components of angiogenesis, the newborn vessels of the developing embryo specialize further and differentiate into arteries, veins, and capillaries. Our results presented here not only identify the phenotype of delayed embryonic development in zebrafish caused by *fars2* deficiency but also preliminarily suggest that this phenotype may be caused by impaired angiogenesis.

Although angiogenesis is an energy-intensive process, glycolysis is the primary energy-producing mechanism in vascular ECs, a feature that is attributable to their special physiological distribution and high levels of exposure to oxygen (27, 28, 63). Historically, the role of mitochondrial respiration in angiogenesis has been overlooked, that is, until the discovery of the essential role of mitochondrial fatty acid and amino acid oxidation pathways in angiogenesis (34). A growing body of evidence suggests that, by acting as important organelles that sense ambient oxygen concentrations and generate energy, the mitochondria play an integral role in controlling metabolism and in regulating the proliferation and survival of ECs during angiogenesis. The mutation of mitochondrial tRNA and aberrant tRNA metabolism induce mitochondrial dysfunction, leading to apoptosis and impaired angiogenesis in HUVECs (64). The mitochondrial permeability transition pore also plays a role in regulating mitochondrial metabolism in ECs and in the maintenance of vascular integrity (65). In addition, mitochondrial dynamics (44) and mitochondria–endoplasmic reticulum contacts (66, 67) are critical for the regulation of angiogenesis and vascular remodeling. In our current study, we found that the impairment of HUVEC proliferation, migration, and tube formation by *FARS2* deficiency was caused by abnormal mitochondrial respiratory function.

The hierarchical organization of ECs into tip cells (leading role) and stalk cells (trailing role) is required by angiogenesis. Tip cells lead the sprouts toward the signaling sources of angiogenesis in tissues, and the tip cells are followed by stalk cells, which elongate the sprout (24, 68). These processes are orchestrated by a complex molecular network, like Notch, Wnt, and VEGF/VEGFR. In tip cells, the activation of VEGFR2 induced the expression of DLL4 in response to VEGF from the signaling source (69). Then, DLL4 activates Notch in stalk cells to restrict branching. Studies in zebrafish and mice reveal that Notch is essential for restricting EC behavior to tip cells, reflected in the excessive sprouting of arteries in the absence of the Notch and the damage of angiogenesis in the activation of the Notch (70, 71). In ECs, Wnt signals could induce a Notch-like phenotype in a reciprocal feedback role, characterized by vascular remodeling and branching defects (39). Studies in mice reveal that Wnt is also required for angiogenesis, reflected in vascular defects after gene-inactivation of the Wnt genes (72). In our study, the activation of Notch and the inhibition of Wnt caused by *FARS2* deficiency might damage angiogenesis by breaking the determination of EC fate and disrupting the signaling system in ECs. In addition, we detected that the transcript of *dll4* and *notch1a* had no significant changes in zebrafish, which was inconsistent with the results of HUVECs. However, the regulation of angiogenesis *in vivo* is an extremely complex process involving various network pathways. In *fars2* deficiency in zebrafish, the upregulation of *hey2* and *notch1b* could partially indicate the activation of Notch signaling pathway (73, 74), but no changes in *dll4* and *notch1a* were potentially due to the crosstalk with other signaling pathways, like VEGF/VEGFR (75). Moreover, we are eager to explore the specific molecular mechanisms involved in these processes during future research.

Expanding research into brain science has produced a large amount of evidence showing that angiogenesis plays a neurotrophic role in neurodegenerative disorders such as Alzheimer's disease. The relationship between cerebrovascular abnormalities and cognitive decline is supported by the fact that Alzheimer's disease brains display vascular pathology, with microvasculature changes occurring before cognitive decline and preceding neurodegenerative changes (76–78). In addition, there is sufficient evidence to suggest that vascular endothelial growth factor-based gene or protein therapies could be used to treat amyotrophic lateral sclerosis patients (79). Although mutations in the *FARS2* gene have a strong association with neurological diseases, the relationship between neural microvascular networks and disease phenotypes in patients with these mutations has not been characterized. Our study may provide new insights into the progression of neurovascular diseases and the diagnosis and treatment of *FARS2* mutation-related genetic diseases.

In summary, using *in vivo* and *in vitro* knock-down models, we report that *FARS2* is essential for angiogenesis. In this study, we focused on elucidating the phenotypes associated with angiogenic defects caused by *FARS2* deficiency. However, the specific molecular mechanisms linking cardiovascular system defects to the impairment of mitochondrial respiratory function due to *FARS2* deficiency have not been investigated thoroughly. In addition, the interaction between the pathogenesis of neurodegenerative diseases and impairment of angiogenesis caused by *FARS2* defects requires further exploration.

## Data Availability Statement

The datasets presented in this study can be found in online repositories. The names of the repository/repositories and accession number(s) can be found in the article/Supplementary Material.

## Ethics Statement

The animal study was reviewed and approved by the Fourth Military Medical University.

## Author Contributions

BL contributed to the conceptualization, data curation, investigation, statistical analysis, visualization, and writing of the original draft. KC and FL contributed to the conceptualization, project administration, methodology, software, editing, and writing of the original draft. JZ contributed to the conceptualization, data collection, and writing of the original draft. XC contributed to the conceptualization, methodology, and writing of the original draft. TC, QC, YY, and WH contributed to the methodology, data collection, data validation, formal analysis, and resources. YW and LW contributed to the conceptualization, project administration, and writing (editing). All authors contributed to the article and approved the submitted version.

## Conflict of Interest

The authors declare that the research was conducted in the absence of any commercial or financial relationships that could be construed as a potential conflict of interest.

## Publisher's Note

All claims expressed in this article are solely those of the authors and do not necessarily represent those of their affiliated organizations, or those of the publisher, the editors and the reviewers. Any product that may be evaluated in this article, or claim that may be made by its manufacturer, is not guaranteed or endorsed by the publisher.

## Abbreviations

mtARSs: mitochondrial aminoacyl-tRNA synthetases
FARS2: mitochondrial phenylalanyl-tRNA synthetase
siRNAs: small interference RNAs
HUVECs: human umbilical vein endothelial cells
ARSs: aminoacyl-tRNA synthetases
mtDNA: mitochondrial DNA
OXPHOs: oxidative phosphorylation system
Phe: Phenylalanine
mt-tRNA^Phe^: mitochondrial phenylalanyl-tRNA
CNS: central nervous system
CVDs: cardiovascular diseases
ECs: endothelial cells
MOs: Morpholinos
ISVs: intersegmental vessels
DLAVs: dorsal longitudinal anastomotic vessels
PAVs: parachordal vessels
CVP: caudal vein plexus
hpf: hours post-fertilization
OCR: oxygen consumption rate
ROS: reactive oxygen species.
