# Supplementary material for: Developmental Angiogenesis Requires the Mitochondrial Phenylalanyl-tRNA Synthetase
Source: Front Cardiovasc Med. 2021 Sep 1;8:724846. doi: 10.3389/fcvm.2021.724846 (PMC8440837; doi:10.3389/fcvm.2021.724846)
Supplement: Supplementary file 1 [file Data_Sheet_1.docx]

Supplementary Material

**Figure S1. The establishment of a *fars2* knock-down zebrafish model.**

**A** Schematic illustration showing the target positions (red bars) of the two morpholinos (ATG-MO and E3I3-MO) used to generate *fars2* knock-down zebrafish.

**B** RT-PCR analyses of *fars2* transcripts from zebrafish embryos injected with a non-specific control MO or the *fars2*-specific E3I3-MO. Analyses were performed at 2 days post-fertilization (dpf).

**C** The expression levels of *fars2* measured by qRT-PCR in zebrafish embryos injected with a non-specific control MO or the *fars2*-specific MOs. Samples were collected at 2 dpf after introduction of 4 ng of the indicated MOs at the one-cell stage (n=30). ***P<0.001.

**Figure S2. Establishment of a *FARS2* knock-down cell model *in vitro*.**

**A** A qRT-PCR analysis of *FARS2* gene expression in HUVECs 48 hours after transfection with control (siCtrl) or *FARS2*-specific (si-*FARS2*) siRNAs.

**B** Western blotting analysis of FARS2 protein levels in HUVECs 48 hours after transfection with the indicated siRNAs. The expression level of GAPDH was used as a loading control.

**C** Quantification of the data shown in B. Measurements were made in triplicate (mean and SEM), and results are indicative of three independent experiments. **P<0.01, ***P<0.001, ****P<0.0001 versus siCtrl.

**Table S1.** List of oligonucleotides used for zebrafish and human qRT-PCR analyses

| **Species** | **Gene** | **Forward Primer (5’-3’)** | **Reverse Primer (5’-3’)** |
| --- | --- | --- | --- |
| zebrafish | *fars2* | CGAACGCCTCAAAAGCAG | GAAGTAACAGTCCACCCAGC |
|  | *ef1α* | GGAAATTCGAGACCAGCAAATAC | GTCGTCCAGCAGAGAATAGAAG |
|  | *dll4* | AGGCCTGGCACTCACCTTACTC | CACCCCAGCCCTCTTTACAGTT |
|  | *notch1a* | GCCGCAGATGCAGGGCAATGAAGT | GAGGGCAGGCAGGGCTGGTAGAGG |
|  | *notch1b* | TGTGAACCAACTGCAGACG | GTTGGCAGAAGTGATCAAACG |
|  | *hey2* | CGGCTTCCGGGAGTGTCTGACT | TCCCCACGGTCGGTATGGTTTA |
|  | *dkk1b* | TCGCCCATGAAAACTCTACTG | TGGACCAAAAGTGACGAGC |
|  | *wnt8a* | CTCTACTCACAAAGGCTTGAGAA | CAAGACTGCAGTTTCTGGTTAAAG |
|  | *wnt9a* | CGCTACAAAATGCTGGATGG | TCAGTGGCAGAATGGACAG |
|  | *β-catenin* | CTGCACATTCTAGCCAGAGAC | CCTTATCCTGAGCCAGTTCAC |
|  | *gsk-3β* | ATCTTAATCCCCGCTCATGC | CAGGTTGAGGTGTTAGAGGC |
|  | *axin1* | GACATGGAGAGGAACCAGAAG | ATGACCCTGAGCTTTCTTGG |
|  | *axin2* | CTTACCCTCGGACACTTCAAG | CCCTCATACATTGGCAGAACTG |
|  | *lef1* | AATTTCTATCCCCTTTCCCCG | TGCTCCTGTTTCACCTGTG |
| Human  (HUVECs) | *FARS2* | CTTACCAGGCTCATGGCACA | AGCACCAGCTGAATTGACCA |
|  | *GAPDH* | CTGGGCTACACTGAGCACC | AAGTGGTCGTTGAGGGCAATG |
|  | *DLL1* | TGTGACGAGTGTATCCGCTAT | GTGTGCAGTAGTTCAGGTCCT |
|  | *DLL3* | CACTCCCGGATGCACTCAAC | GATTCCAATCTACGGACGAGC |
|  | *DLL4* | GCCCTTCAATTTCACCTGGC | CAATAACCAGTTCTGACCCACAG |
|  | *NOTCH1* | TGGACCAGATTGGGGAGTTC | GCACACTCGTCTGTGTTGAC |
|  | *NOTCH2* | GATCACCCGAATGGCTATGAAT | GGGGTCACAGTTGTCAATGTT |
|  | *NOTCH3* | CGTGGCTACACTGGACCTC | AGATACAGGTGAACTGGCCTAT |
|  | *NOTCH4* | GTGAACGTGATGTCAACGAG | ACAGTCTGGGCCTATGAAACC |
|  | *DKK1* | CCTTGAACTCGGTTCTCAATTCC | CCTTGAACTCGGTTCTCAATTCC |
|  | *β-catenin* | CATCTACACAGTTTGATGCTGCT | GCAGTTTTGTCAGTTCAGGGA |
|  | *AXIN2* | TACACTCCTTATTGGGCGATCA | TTGGCTACTCGTAAAGTTTTGGT |
|  | *AXIN1* | GGTTTCCCCTTGGACCTCG | CCGTCGAAGTCTCACCTTTAATG |

**Table S2.** List of target oligonucleotide sequences used for FARS2 knock-down in HUVECs

| **siRNAs** | **Target sequence (5’-3’)** |
| --- | --- |
| si-*FARS2* #1 | GGAAAAGGTTGATCTCATA |
| si-*FARS2* #2 | GGACAACTATTACCTGAAT |
| si-*FARS2* #3 | GGCTAGCCATGATCCTCTA |
