# Supplementary figures and images for: Developmental Angiogenesis Requires the Mitochondrial Phenylalanyl-tRNA Synthetase
Source: Front Cardiovasc Med. 2021 Sep 1;8:724846. doi: 10.3389/fcvm.2021.724846 (PMC8440837; doi:10.3389/fcvm.2021.724846)

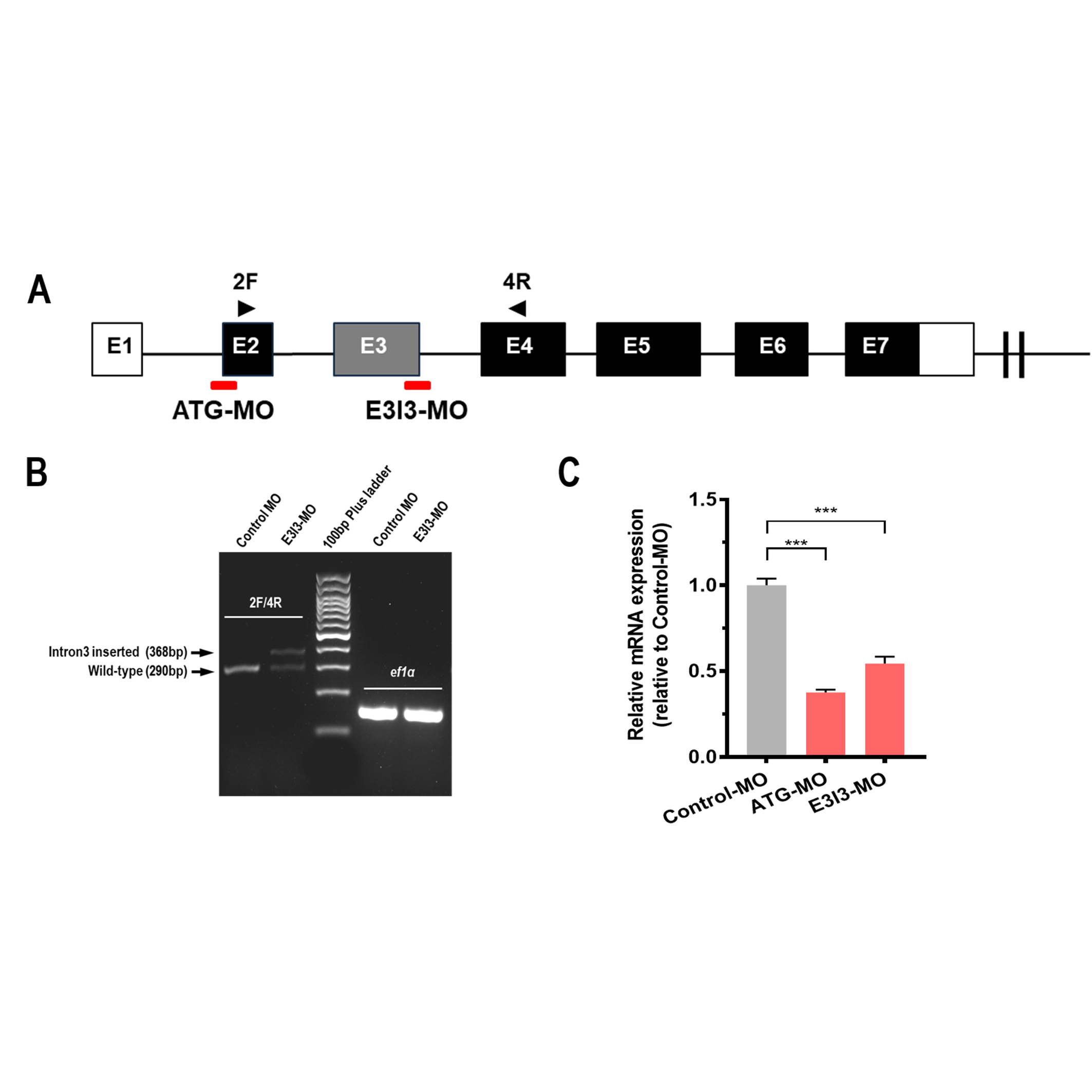

Supplement: Supplementary file 2 [file Image_1.TIF]

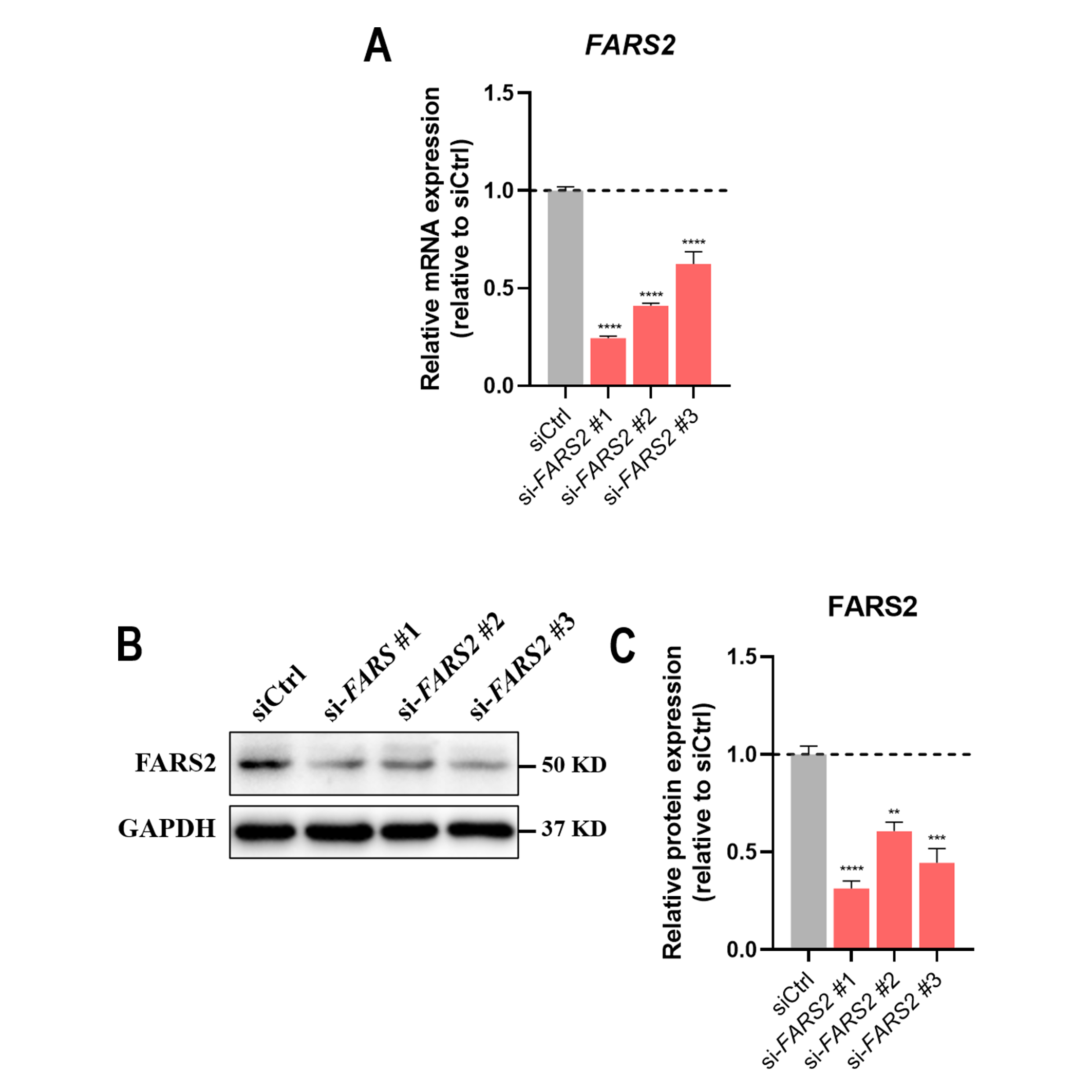

Supplement: Supplementary file 3 [file Image_2.TIF]
